# Nontraumatic Anterior Thigh Pain in a NCAA Lacrosse Athlete: A Case Report and Description of a Rectus Femoris Degloving Injury

**DOI:** 10.1155/2019/2735309

**Published:** 2019-11-18

**Authors:** Paul A. Krebs, James R. Borchers, Patrick Brayfield

**Affiliations:** ^1^Premier Health, Department of Family Medicine, Wright State University Boonshoft School of Medicine, 2400 Miami Valley Drive Suite 160 Dayton, Ohio 45459, USA; ^2^The Ohio State University Wexner Medical Center Jameson Crane Sports Medicine Institute, 2835 Fred Taylor Drive, Columbus, Ohio 43202, USA; ^3^The Ohio State University, Athletic Training Staff, 615 Irving Schottenstein Dr., Columbus, OH 43210, USA

## Abstract

A 21-year-old female lacrosse player complained of anterior thigh pain with no known mechanism of injury and failed to improve with conservative therapy. An MRI was obtained showing a closed degloving injury of the rectus femoris, an injury only previously reported in a small case series of soccer players. After a brief period of rest, she was progressed conservatively through therapy and did well, with progression back to the level of competition at 56 days. This case highlights a rare injury not previously described in sports outside of soccer and is the first case described in a female athlete. In addition, the discussion of this case focuses on the unique anatomy of the rectus femoris.

## 1. Introduction

Injuries to the quadriceps muscles are common in athletics. Certain sports such as soccer may show higher rates of adductor injuries compared to quadriceps injuries, but in aggregate studies of all sports, quadriceps injuries appear to be the second most common type of lower extremity muscle injuries behind hamstring injuries [1–4]. Quadriceps injuries include strains, tears, contusions, and hematomas. The purpose of this report is to highlight a case of anterior thigh pain that presented with symptoms similar to a quadriceps strain, and then to discuss closed degloving injuries of the rectus femoris, a rare injury to the quadriceps that may be overlooked in clinics.

The patient was informed that the details of her case would be submitted for print and electronic publication and gave informed consent.

## 2. Case Presentation

**Figure 1:**
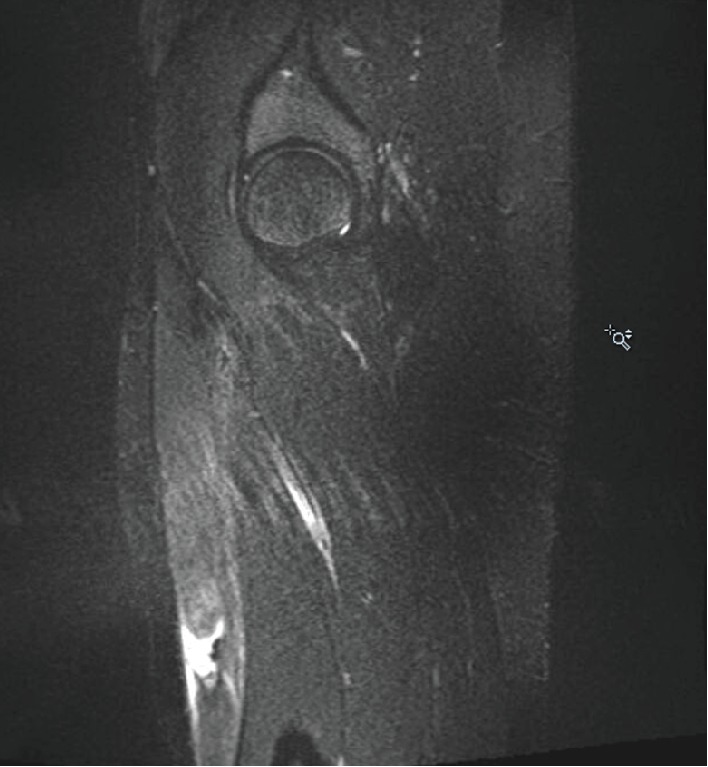
T2 STIR sagittal MRI image of the right femur without contrast demonstrating an acute degloving injury of the right rectus femoris.

**Figure 2:**
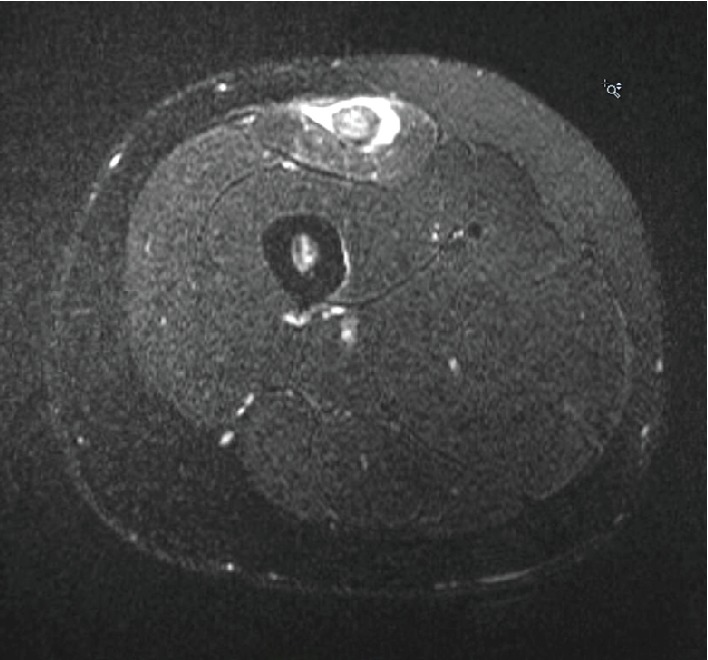
TSE T2 axial MRI image of the right femur without contrast demonstrating an acute degloving injury of the right rectus femoris.

A 21-year-old NCAA Division 1 Lacrosse player presented to the training room with right anterior thigh pain of 4-5 weeks duration. Onset was gradual, occurring in the spring lacrosse season and starting during training and practice. She did not report any new activity or specific mechanism of injury leading to the pain. She had no history of surgery or major injury to the region. She had previously been seen by the athletic trainer at the onset of pain, described initially as a soreness in her anterior thigh of gradual onset at practice, and started on conservative management for a presumed quadriceps strain. This initial management consisted of modified activities at practice, rehabilitation therapy in the training room, NSAIDS, ice, cupping, and needling. Despite some initial improvement with these treatments, symptoms increased with gradual return to lacrosse. The pain was now throbbing, worse after games, occurring at night, waking her up from sleep, and exacerbated when her leg would hang over the edge of the chair. On exam, she had full active and passive range of motion and strength of the hip as well as the knee. She had tenderness over the right anterior mid-proximal thigh, extending over and area of approximately 10 cm. The tenderness was worse medially than laterally with no palpable defect in the muscle, and she had a positive fulcrum and hop test. Her neurovascular exam was intact. An X-ray of the femur did not reveal any abnormalities. An MRI was ordered to rule out a stress fracture and to evaluate the quadriceps muscle further. The MRI showed an acute complete disruption of the inner bipennate muscular component of the right rectus femoris from the more superficial unipennate muscular component with retraction of the inner component, consistent with an acute degloving injury. No abnormality was noted in the osseous structure. These findings are highlighted in Figures 1 and 2. Given these findings of a closed degloving injury of the rectus femoris, the decision was made to discontinue all lacrosse activities and start a period of rest. After 2 weeks of rest with only a gentle range of motion exercises, her pain had improved. She progressed through therapy conservatively as the lacrosse season had just ended, starting with isometrics, and then progressing as tolerated to dynamic exercises followed by lacrosse-specific drills and exercise. By six weeks (42 days), she felt great and was cleared for full activity in therapy/progression to sport with estimated return to full competition at eight weeks (56 days) had it not been the offseason. The patient was able to participate in all offseason activities and had no setbacks or recurrence of symptoms the following season, returning fully to the level of play prior to injury.

## 3. Discussion

Injuries to the quadriceps muscles are common in athletics, with the rates of injury reported to be higher in women and occurring more frequently in competition [1, 5]. Among NCAA athletes, quadriceps strains, the most common type of quadriceps injury, are reported to occur at a rate of 1.07/10,000 athlete exposures (AEs). Examining different sports, women's soccer has the highest overall reported rate at 5.61/10,000 AEs [1]. Of the quadriceps muscles, the rectus femoris is the most commonly injured [6].

The anatomy of the rectus femoris is unique compared to the other quadriceps muscles. In addition to having actions on both the hip and knee joint, the anatomy of the muscle and proximal tendons creates a “muscle within a muscle” [7]. Cadaveric studies and MRI studies demonstrate an intramuscular bipennate structure and central aponeurosis arising from the indirect tendon off the superior acetabular ridge, surrounded by a unipennate superficial portion arising from the direct tendon off the anterior inferior iliac spine [4, 7, 8]. This anatomy creates the possibility for the type of injury highlighted in this case report, termed a closed degloving injury, where the inner bipennate muscle tears and retracts within the outer unipennate muscle [9]. The anatomy of the quadriceps demonstrates two different muscle tendon structures that can be seen in the human body, unipennate and bipennate structures. These terms describe the arrangement of the muscle fibers around the tendon. A unipennate muscle is one where all the fibers originate from one side of the tendon, while a bipennate muscle has muscle fibers originating from two sides of a tendon [4].

Central tendon and muscle injuries have been found to have prolonged recovery when compared to injuries to the periphery [6, 10]. While previous literature describes central tendon and muscle injuries, including proximal tendon injuries leading to “bull's eye injuries” [6, 10], degloving injuries seen on MRI have only been described once, in a case series of 8 male athletes [9]. The case presented is unique in that it is the first reported case in a female and nonsoccer athlete. Additionally, there was no known incident or event precipitating this injury. In the previous case series, the history of 7 of the 8 athletes was described, with all injuries occurring during a soccer practice or game, with the most common mechanism being kicking (4 of 7) [9]. Similar to 4 of the athletes in the previous case series, the athlete in this case report was able to initially continue to play with symptoms. The time until return to competition from diagnosis in the athlete in this case report was 56 days compared to the 38.7 day average in the previous case series [9]. This may be explained by the conservative approach to therapy for the athlete as it was the offseason. Other characteristics of the lesion, including the distance between the acetabular rim and the proximal portion of the injury (17.9 cm vs 15.5 cm (range 11.3-20.3 cm)) and the degree of retraction (1.1 cm vs 1.2 cm (range 0-3.5 cm)) were similar between this case report and the previous case series, respectively [9].

A thorough understanding of the unique anatomy of the rectus femoris first elucidated in the 1990s by Hughes and Hasselman is important to understanding the range of injuries that can occur within the quadriceps muscles [7, 8]. This case report of a female lacrosse player with a degloving injury of the rectus femoris highlights the anatomy of the muscle, the potential for unique injury patterns, and need for further investigation when patient's recovery times are prolonged despite appropriate therapy.
